# Supplementary material for: A Bayesian Approach to Correcting the Attenuation Bias of Regression Using Polygenic Risk Score
Source: bioRxiv. 2023 Nov 28:2023.11.27.568907. Preprint. [Version 1] doi: 10.1101/2023.11.27.568907 (PMC10705229; doi:10.1101/2023.11.27.568907)
Supplement: 1 [file NIHPP2023.11.27.568907V1-supplement-1.pdf]

bioRxiv preprint doi: https://doi.org/10.1101/2023.11.27.568907; this version posted November 28, 2023. The copyright holder for this preprint (which was not certified by peer review) is the author/funder, who has granted bioRxiv a license to display the preprint in perpetuity. It is made available under aCC-BY 4.0 International license.

Supplementary materials

	**No correction**	**Correction with measurement error**
HDL~LDL	0.011	0.052
HDL~TC	0.142	0.015
HDL~log(TG)	0.335	0.012
LDL~HDL	0.053	0.208
LDL~TC	0.571	0.006
LDL~log(TG)	0.209	0.170
TC~HDL	0.206	0.197
TC~LDL	0.606	0.049
TC~log(TG)	0.226	0.154
TG~HDL	0.296	0.031
TG~LDL	0.225	0.086
TG~TC	0.244	0.016
Height~BMI	0.030	0.019

Supplementary Table 1. Linear regression coefficients for pairs of continuous traits.

bioRxiv preprint doi: https://doi.org/10.1101/2023.11.27.568907; this version posted November 28, 2023. The copyright holder for this preprint (which was not certified by peer review) is the author/funder, who has granted bioRxiv a license to display the preprint in perpetuity. It is made available under aCC-BY 4.0 International license.

	**No correction**	**Correction with measurement error**
CAD~HDL	0.146	0.132
CAD~LDL	0.232	0.085
CAD~TC	0.193	0.089
CAD~log(TG)	0.181	0.074
AF~HDL	0.006	0.085
AF~LDL	0.074	0.006
AF~TC	0.101	0.026
AF~log(TG)	0.126	0.012
DIA~HDL	0.502	0.135
DIA~LDL	0.092	0.020
DIA~TC	0.137	0.114
DIA~log(TG)	0.446	0.116

Supplementary Table 2. Logistic regression coefficients for binary outcomes.
